# Comparison of the Development and Prognosis in Patients of Hypertriglyceridemic Pancreatitis with and without Diabetes

**DOI:** 10.1155/2021/8895268

**Published:** 2021-12-09

**Authors:** Wenjian Liao, Xiuxiu Niu, Wei Zhang, Xiaobing Liu

**Affiliations:** ^1^Department of Respiratory and Critical Care Medicine, The First Affiliated Hospital of Nanchang University, Nanchang 330006, China; ^2^Department of Endocrinology, HuiDong People's Hospital, Guangdong 516300, China

## Abstract

**Aim:**

To investigate the clinical features and prognosis in patients of hyperlipidemic acute pancreatitis with or without diabetes.

**Methods:**

157 patients with hypertriglyceridemic pancreatitis (HTGP) were included in this study. Patients with a previous history of diabetes were identified in the group of HTGP with diabetes (HTGPD), while patients without a history of diabetes were identified in the group of HTGP. The clinical characteristics and prognosis data of these patients in the two groups were analyzed.

**Results:**

Multivariate Cox regression analysis showed that age, body mass index, glycated serum protein (GSP), and Acute Physiology and Chronic Health Evaluation (APACHE) II score were significantly associated with mortality in patients with HTGP. The mortality was significantly higher in the HTGPD group than in the HTGP group (*p* < 0.001). Compared to patients of HTGP, those of HTGPD had older age of onset, higher blood glucose levels, and higher GSP levels on admission. Electrocardiograms showed that patients of HTGPD had a significantly higher risk of heart ischemia than those of HTGP (*p* < 0.05). Patients of HTGPD had higher APACHE II scores than those of HTGP (*p* < 0.001). Single-factor analysis showed that higher triglyceride levels, GSP, LDL, and previous history of diabetes were associated with HTGP recurrence.

**Conclusions:**

Clinicians should be alert to patients of HTGP with diabetes. Diabetes is an important risk factor for HTGP and hyperglycemia may affect the development and prognosis of HTGP.

## 1. Introduction

Diabetes has become endemic in China. Based on the most recent data, 12.8% of the population in China has diabetes, while as much as 35.2% of the population has prediabetes [1]. Patients with type 2 diabetes are prone to suffer hypertriglyceridemia (HTG) and biliary calculi simultaneously [2]. Acute pancreatitis (AP) is an important cause of morbidity and mortality worldwide with an increasing annual incidence. The main risk factor for AP in China is biliary pancreatitis, while the rate of AP induced by HTG is increasing [3]. Poorly controlled diabetes, obesity, and high-fat diets can contribute to elevated triglyceride (TG) levels and then provoke AP. A Japanese observational study found an increased risk of acute pancreatitis in patients with type 2 diabetes mellitus [4]. In other words, if self-monitoring of blood glucose in patients with a long history of diabetes is poor, especially for glycated serum protein (GSP) and glycosylated hemoglobin A1c (HbA1c), the continuously high blood glucose can increase patients' risk of suffering AP. Through evaluating the clinical features and prognosis in patients of hypertriglyceridemic pancreatitis with diabetes (HTGPD) in this study, we hope to provide certain evidence to guide HTGPD's prevention and control.

## 2. Methods

This was a retrospective study over 3 years from January 2010 to December 2012. 157 patients with HTGP were diagnosed and treated at The First Affiliated Hospital of Nanchang University. The diagnosis of HTGP required (1) diagnosis according to American guidelines of the diagnosis and treatment of AP [5]; (2) serum TG ≥ 5.65 mmol/L [6]. Patients with a previous history of diabetes were identified in the group of HTGP with diabetes (HTGPD), while patients without a history of diabetes were identified in the group of HTGP. General information such as age, gender, course of diabetes, and previous history of chronic diseases such as liver disease, heart disease, kidney disease, and lung disease was recorded. On admission, body temperature and blood pressure were measured; an abdominal computed tomography (CT) scan was performed; plasma glucose, GSP, electrolyte, procalcitonin, C-reactive protein (CRP), serum amylase, and ketone bodies were determined using regular biochemical assays. Body mass index (BMI), Acute Physiology and Chronic Health Evaluation (APACHE) II score, bedside index for severity in AP score, and CT severity index score were calculated, the APACHE-II score was calculated during the first 24 h after admission; myocardial ischemia was identified on an electrocardiogram (a horizontal or downsloping ST-T segment with a reduction > 0.1 mV as well as flattened or inverted T waves). The study protocol has been approved by the Ethics Committee of the First Affiliated Hospital of Nanchang University.

## 3. Statistical Analysis

Normally distributed data were expressed as means ± SD, whereas variables with a skewed distribution were reported as median (interquartile range) and log-transformed to approximate normality before the analysis. Enumeration data were expressed as a positive rate or percentage. Student's *t*-test, the Mann-Whitney *U*-test, and the chi-square test were used to describe patients' baseline characteristics. Survival curves were plotted according to the Kaplan-Meier method, and their statistical differences were analyzed by the log-rank test. Cox proportional hazard regression was used to test the association between clinical prognostic factors and mortality in patients with HTGP. Variables that showed an indication of prognostic influence in univariate Cox regression analysis were included in multivariate Cox regression analysis using the backward elimination method. All the differences in the clinical features were analyzed between HTGP and HTGPD. Logistic regression was used to analyze the risk factors of HTGP recurrence. All statistical analyses were performed using the SPSS Statistical Package (version 20.0). Values of *p* < 0.05 were considered statistically significant.

## 4. Results

### 4.1. Analysis of General Clinical Data

This study included 104 patients with HTGP alone (HTGPA) and 53 patients with HTGPD. The mean age of the total 157 patients in this study was 47.9 ± 12.7 (range, 24-79) years; there were more males than females (105 [67.9%] vs. 52 [33.1%], respectively). Among them, 104 had HTGPA, and 53 were HTGPD. All 53 diabetic patients were of type 2. The average diabetic course was 4.0 ± 1.7 years. All of 157 patients had characteristic CT imaging changes of AP. Among the 53 patients with HTGPD, 48 took oral antidiabetic drugs, two underwent diet and exercise intervention, and three were under no treatment. In the HTGPA group, 21 (20.2%) subjects had ischemic heart disease, eight (7.7%) had chronic obstructive pulmonary disease (COPD), and 30 (28.8%) had hypertension. In the HTGPD group, 12 (22.6%) subjects had ischemic heart disease, four (7.5%) had the COPD, and 18 (33.9%) subjects had hypertension. The mean APACHE II score of the HTGPD group (7.1 ± 3.5) was significantly higher than that of the HTGPA group (5.1 ± 3.2) (*p* < 0.001). Disease incubation period occurred slightly later in the HTGPD group than in the HTGPA group. The patients in the HTGPA group had a shorter time from first symptoms to admission, and most patients in this group went to the hospital for abdominal pain. Almost all patients in the HTGP group had obvious abdominal pain, while only 81.0% of the subjects in the HTGPD group had abdominal pain. All patients had a higher than normal BMI (Table 1).

### 4.2. Analysis of Laboratory Data

There was no obvious difference in temperature or blood pressure in patients of the HTGPD and HTGPA groups on admission. Patients in the HTGPD group had a slightly higher mean glucose level (16.1 ± 7.1 mmol/L) than those in the HTGPA group (8.6 ± 3.7 mmol/L), (*p* < 0.001). The mean GSP of the HTGPD group (2.8 ± 0.6 mmol/L) was higher than that in the HTGPA group (1.9 ± 0.4 mmol/L). The mean low-density lipoprotein cholesterol (LDL-C) level of patients in the HTGPD group (2.0 ± 1.2 mmol/L) was higher than that of patients in the HTGPA group (1.1 ± 0.8 mmol/L). The mean CRP level of the HTGPD group (229.2 ± 108.7 mg/L) was higher than that of the HTGPA group (162.7 ± 109.7 mg/L). Electrocardiogram findings revealed that patients with HTGPD had a significantly higher risk of heart ischemia than patients with HTGPA (39/53; 73.6%; vs. 27/104; 25.9%, respectively; *p* < 0.05; Table 1).

### 4.3. Analysis of Prognosis and Risk Factors

**Figure 1:**
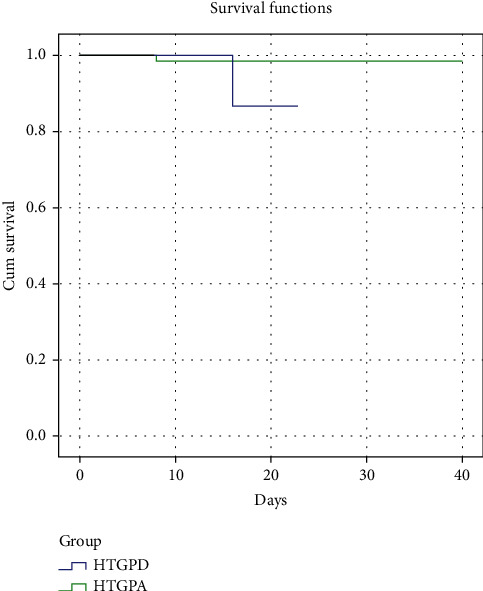
Survival curves showed a significant survival difference between the HTGPD group and HTGPA group via the log-rank test.

Of the patients with recurrent acute pancreatitis (28/157; 17.8%), 18 had HTGPD, meaning that the recurrence rate of patients with HTGPD (18/53; 34.0%) was higher than that of patients with HTGPA (10/104; 9.6%; *p* < 0.001). Univariate analysis showed that triglyceride levels and previous history of diabetes were associated with HTGP recurrence. On stepwise logistic regression analysis, HTG and previous history of diabetes were associated with HTGP recurrence (all subjects; odds ratio > 5; Table 2). In-hospital mortality rate was significantly higher in the HTGPD group (2/53, 3.8%) than in the HTGPA group (1/104; 0.96%; *p* < 0.001). Survival curves showed a significant survival difference between the HTGPD group and HTGP group via the log-rank test (Figure 1), while multivariate Cox regression analysis showed that age, BMI, GSP, and APACHE II score were significantly associated with HTGP-related mortality (Table 3).

## 5. Discussion

The prevalence of diseases associated with obesity and diabetes has increased in recent years and contributes substantially to healthcare costs and mortality rates [7]. In China, the incidence of HTGP continues to increase annually as living standards improve. Related research has confirmed that this is due to the epidemic of obesity and diabetes [8]. The HTGP-related frequency of severe AP and organ dysfunction and recurrence and mortality rates were significantly higher than those of biliary-induced pancreatitis [9]. GSP can indicate the average blood glucose level within the preceding 2–3 weeks. It is important for us to understand the average blood glucose level before AP onset. AP is a clinical emergency, so the observation of changes within the body before its onset is meaningful for its diagnosis and treatment. We chose GSP rather than HbA1c since it can better reflect the effect of elevated blood glucose on HTGP.

Most of the patients with HTGP were accompanied by hyperglycemia [10]. Analysis of laboratory and clinical data revealed that hyperglycemia affects AP severity as well as recurrence and mortality rates. Related studies found that patients with the combination of obesity, diabetes, and excessive alcohol consumption are prone to developing extremely high TG values [11]. Certainly, HTG was the most important risk factor for HTGP, especially during pregnancy [12].

In the current study, LDL-C levels of patients with HTGPD were higher than those of patients with HTGPA. A high LDL-C contributes to an elevated cardiovascular risk, thus increasing mortality rates of patients with HTGPD. Controlled LDL-C levels facilitate a substantial reduction in cardiovascular risk [13]. As is shown in Table 1, indices of inflammation such as white blood cell count and CRP level differed obviously between patients with HTGPA and those with HTGPD. Related studies found that inflammation contributes to the pathogenesis of type 2 diabetes and that anti-inflammatory therapies are potential treatments for obesity-related insulin resistance and glucose intolerance [14, 15]. We also have some other doubts whether the inflammatory response in AP is associated with hyperglycemia and whether the better detection and control of blood glucose levels will reduce the incidence of AP. These questions require further observation and study. The main limitation of our study was its retrospective design and data from previous cases; they may be related to disease history which is not complete and analysis of prone to bias; as some parameters were lacking, such as serum c-peptide levels and the intervention of all kinds of drugs, they may be associated with AP. Prospective studies should be performed to confirm our results.

In conclusion, physicians should be alert to HTGPD. Diabetes is an important risk factor for HTGP, and hyperglycemia may affect the development and prognosis of HTGP.

## Figures and Tables

**Table 1:** Comparison of the clinical features of patients with hypertriglyceridemic pancreatitis with or without diabetes.

Clinical characteristics	HTGPA (*n* = 104)	HTGPD (*n* = 53)	*F*(*χ*)	*p* value
Age (year)	46.3 ± 10.1	51.9 ± 12.5	6.18	**0.014**
Sex (male/female)	73/31	32/21	1.38	0.241
Alcohol intake history (yes/no)	48/59	26/24	0.07	0.816
Time from first symptoms to admission (d)	1.8 ± 1.9	4.4 ± 2.1	1.71	0.194
Abdominal pain (yes/no)	101/6	43/7	2.56	0.111
Emesis (yes/no)	48/59	26/24	0.07	0.816
Temperature (°C)	36.9 ± 0.6	37.0 ± 0.5	3.54	0.062
Systolic pressure (mmHg)	127.0 ± 18.7	132.3 ± 23.3	2.51	0.115
Diastolic pressure (mmHg)	85.5 ± 12.9	85.6 ± 14.2	0.05	0.817
BMI (kg/m^2^)	25.0 ± 2.1	25.2 ± 2.3	0.09	0.755
Blood glucose on admission (mmol/L)	8.6 ± 3.7	16.1 ± 7.1	82.04	**<0.001**
GSP on admission (mmol/L)	1.9 ± 0.4	2.7 ± 0.6	151.61	**<0.001**
Triglyceride (mmol/L)	7.1 ± 10.9	9.0 ± 6.1	0.95	0.333
Cholesterol (mmol/L)	5.2 ± 2.5	6.1 ± 2.3	3.41	0.067
Low-density lipoprotein cholesterol (LDL-C) (mmol/L)	1.1 ± 0.8	2.0 ± 1.2	25.29	**<0.001**
Serum calcium (mmol/L)	2.0 ± 0.4	2.1 ± 0.2	1.22	0.272
C-reactive protein (mg/L)	162.7 ± 109.7	229.2 ± 108.7	11.70	**0.001**
Procalcitonin (ng/mL)	2.9 ± 8.4	4.4 ± 8.9	0.46	0.498
White blood cell (×109/L)	12.3 ± 5.7	10.7 ± 3.2	3.51	0.063
Ischaemia in electrocardiogram (yes/no)	27/77	39/14	72.28	**<0.001**
Length of hospitalization (d)	11.0 ± 8.5	11.9 ± 5.1	1.11	0.294
Recurrence (yes/no)	10/94	18/35	57.69	**<0.001**
(APACHE) II score (score)	5.1 ± 3.2	7.1 ± 3.5	0.79	**<0.001**
BISAP (score)	1.3 ± 0.8	1.1 ± 0.9	0.11	0.233
CTSI (score)	3.4 ± 1.4	3.6 ± 2.0	0.07	0.313
Death (yes/no)	1/103	2/51	57.69	**<0.001**

Statistically significant correlations (*p* < 0.05) are shown in bold font.

**Table 2:** Factors of hypertriglyceridemic pancreatitis recurrence on stepwise logistic regression analysis.

Factors	*β*	SE	OR	*p*
Hypertriglyceridemia (HTG)	1.683	0.623	5.411 ± 1.91	0.017
Previous history of diabetes	-1.005	0.610	5.127 ± 1.91	0.032

**Table 3:** Multivariate analysis by Cox proportional hazard regression of risk factors for hypertriglyceridemic pancreatitis-related mortality.

Variables	*B*	Std. error	Beta	*t*	*p*
Age	0.002	0.001	0.194	2.407	0.017
APACHEII	-0.007	0.003	-0.172	-2.061	0.041
BMI	0.013	0.032	0.613	2.310	0.027
GSP	0.044	0.018	0.204	2.504	0.013

## Data Availability

The raw data supporting the conclusions of this article will be made available by the authors, without undue reservation, to any qualified researcher.

## References

[1] Li Y., Teng D., Shi X. (2020). Prevalence of diabetes recorded in mainland China using 2018 diagnostic criteria from the American Diabetes Association: national cross sectional study. *BMJ*.

[2] Bouchaala K., Bahloul M., Bradii S., Kallel H., Chtara K., Bouaziz M. (2020). Acute pancreatitis induced by diabetic ketoacidosis with major hypertriglyceridemia: report of four cases. *Case Reports in Critical Care*.

[3] Yu S., Yao D., Liang X. (2020). Effects of different triglyceride-lowering therapies in patients with hypertriglyceridemia-induced acute pancreatitis. *Experimental and Therapeutic Medicine*.

[4] Urushihara H., Taketsuna M., Liu Y. (2012). Increased risk of acute pancreatitis in patients with type 2 diabetes: an observational study using a Japanese hospital database. *PLoS One*.

[5] Tenner S., Baillie J., DeWitt J., Vege S. S. (2013). American College of Gastroenterology guideline: management of acute pancreatitis. *The American Journal of Gastroenterology*.

[6] Endocrine Society (2013). Endocrine society releases guidelines on diagnosis and management of hypertriglyceridemia. *American Family Physician*.

[7] Go A. S., Mozaffarian D., Roger V. L. (2013). Executive summary: heart disease and stroke statistics--2013 update: a report from the American Heart Association. *Circulation*.

[8] Wang S. Q., Li S. J., Feng Q. X., Feng X. Y., Xu L., Zhao Q. C. (2011). Overweight is an additional prognostic factor in acute pancreatitis: a meta-analysis. *Pancreatology*.

[9] Huang Y. X., Jia L., Jiang S. M., Wang S. B., Li M. X., Yang B. H. (2014). Incidence and clinical features of hyperlipidemic acute pancreatitis from Guangdong, China: a retrospective multicenter study. *Pancreas*.

[10] Lindkvist B., Appelros S., Regnér S., Manjer J. (2012). A prospective cohort study on risk of acute pancreatitis related to serum triglycerides, cholesterol and fasting glucose. *Pancreatology*.

[11] Bessembinders K., Wielders J., van de Wiel A. (2011). Severe hypertriglyceridemia influenced by alcohol (SHIBA). *Alcohol and Alcoholism*.

[12] Takaishi K., Miyoshi J., Matsumura T., Honda R., Ohba T., Katabuchi H. (2009). Hypertriglyceridemic acute pancreatitis during pregnancy: prevention with diet therapy and *ω*-3 fatty acids in the following pregnancy. *Nutrition*.

[13] Chapman M. J., Ginsberg H. N., Amarenco P. (2011). Triglyceride-rich lipoproteins and high-density lipoprotein cholesterol in patients at high risk of cardiovascular disease: evidence and guidance for management. *European Heart Journal*.

[14] Donath M. Y., Shoelson S. E. (2011). Type 2 diabetes as an inflammatory disease. *Nature Reviews. Immunology*.

[15] Gregor M. F., Hotamisligil G. S. (2011). Inflammatory mechanisms in obesity. *Annual Review of Immunology*.

